# Production of Ultrafine PVDF Nanofiber-/Nanonet-Based Air Filters via the Electroblowing Technique by Employing PEG as a Pore-Forming Agent

**DOI:** 10.1021/acsomega.3c05509

**Published:** 2023-10-02

**Authors:** Ali Toptaş, Mehmet Durmuş Çalışır, Ali Kılıç

**Affiliations:** † TEMAG Laboratories, Textile Technol. and Design Faculty, Istanbul Technical University, 34437 Istanbul, Turkey; ‡ Safranbolu Vocational School, Karabuk University, 78600 Karabuk, Turkey; § Faculty of Engineering and Architecture, Recep Tayyip Erdogan University, 53100 Rize, Turkey; ∥ Areka Advanced Technologies LLC, 34467 Istanbul, Turkey

## Abstract

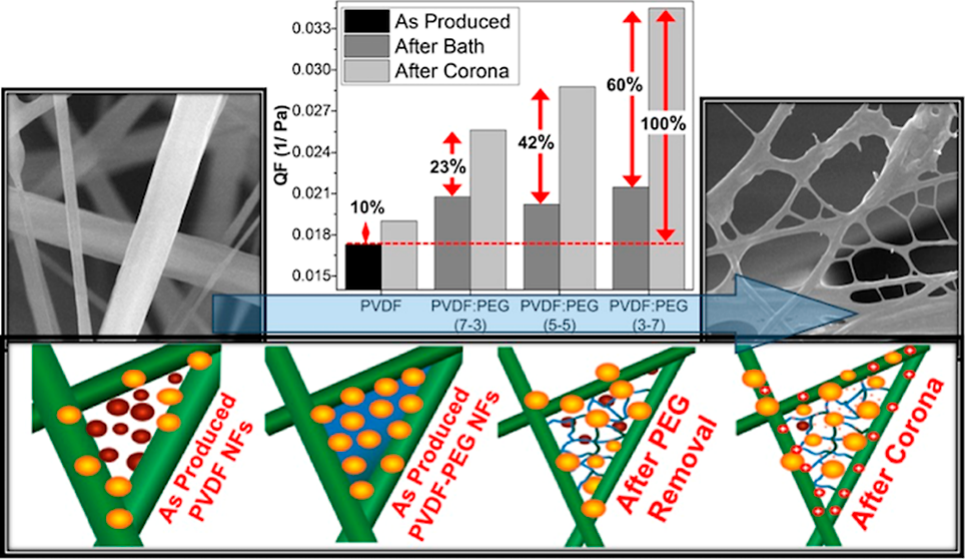

Particles with diameters smaller than 2.5 μm (PM_2.5_) can penetrate the respiratory system and have negative impacts on human health. Filter media with a porous surface and nanofiber/nanonet structure demonstrate superior filtration performance compared to traditional nano- and microfiber-based filters. In this study, nanostructured filters were produced using the electroblowing method from solutions containing different ratios of poly(vinylidene fluoride) (PVDF) and polyethylene glycol (PEG) polymers for the first time. By increasing the water-soluble PEG ratio in PVDF/PEG blend nanofibers and employing a water bath treatment to the produced mat afterward, a more porous fibrous structure was obtained with a lower average fiber diameter. Notably, the removal of PEG from the PVDF/PEG (3–7) sample, which had the highest PEG content, exhibited clustered nanofiber-/nanonet-like structures with average diameters of 170 and 50 nm at the points where the fibers intersect. Although this process resulted in a slight decrease in the filtration efficiency (−1.3%), the significant reduction observed in pressure drop led to a 3.2% increase in the quality factor (QF). Additionally, by exploiting the polarizability of PVDF under an electric field, the filtration efficiency of the nanostructured PVDF filters enhanced with a ratio of 3.6% after corona discharge treatment leading to a 60% improvement in the QF. As a result, the PVDF/PEG (3–7) sample presented an impressive filtration efficiency of 99.57%, a pressure drop (Δ*P*) of 158 Pa, and a QF of 0.0345 Pa^–1^.

## Introduction

The need for clean air is escalating due to population growth and rapid industrial development.^1^ Air pollution, consisting of solid particulate matter (PM), organic substances, and epidemic diseases, poses a significant threat to human health.^2^ Inhaling PM particles can lead to various diseases, including cancer, as they can penetrate the respiratory system.^3^ Personal protective equipment, including air filters, plays a crucial role in purifying inhaled air and removing PM, bacteria, and viruses.^4^ Although traditional air filters composed of micrometer-scale fibers, such as melt-blown, spun-bond, and glass fiber filters, are commonly used,^5^ the air filters must possess nanosized pores to effectively remove fine PM and viruses. Nanofibrous structures are well-suited for this purpose as they offer a cost-effective means of production and have a suitable pore structure.^6^

An ideal air filter should exhibit a high filtration efficiency with a low-pressure drop (Δ*P*). However, in general, achieving high filtration efficiency with small pores leads to increased Δ*P*. Conversely, a filter with large pores results in low filtration efficiency, too.^7–9^ To increase the submicron particle capture efficiency of the traditional filters, electret properties can be imparted to them using methods such as corona discharge,^10,11^ triboelectrification,^12,13^ hydrocharging,^14^ and thermal polarization.^15^ Poly(vinylidene fluoride) (PVDF) is known as a highly stable polymer with electret characteristics and is commonly used in air filtration studies. In a study by Xiao et al.,^16^ 99.97% efficiency was reached at the expense of 137 Pa pressure drop for filter mats composed of electrospun PVDF nanofibers having an average diameter of 30.8 nm. In another work,^17^ sodium dodecyl sulfate was added to control the diameter and uniformity of electrospun PVDF nanofibrous membranes, resulting in PVDF nanofibers with a diameter of 70 nm. Due to the synergistic combination of the slip effect introduced by the nanoscale structure and the electret properties provided by PVDF, a high PM_0.3_ filtration efficiency of 97.40% was achieved at a pressure drop of 51 Pa under an airflow rate of 5.3 cm/s.

Another method to increase the efficiency and reduce the Δ*P* is to use a combination of nano- and microfibers that create bimodal networks.^18,19^ These bimodal networks with a wide range of Knudsen numbers let the air flow with reduced resistance.^20,21^ Bimodal fibers can be created by stacking layers of fibers with different diameters or simultaneously producing fibers of different diameters in a single layer. Zhang et al.^22^ fabricated a layered multimodal filter fabric which consisted of stacked polysulfone microfibers (∼1 μm), polyacrylonitrile (PAN) nanofibers (∼200 nm), and polyamide 6 (PA6) nanowebbed layers (∼20 nm) via electrospinning. The resulting fabric exhibited an impressive filtration efficiency of 99.992% and an Δ*P* of 118 Pa. Gungor et al.^23^ produced PA6 fibers with average fiber diameters (AFD) of 81.5 nm and 1.6 μm by a two-nozzle solution-blowing method using PA6 solutions prepared at different concentrations (7 and 20 wt %). The resulting filters achieved a filtration efficiency of 98.891% and an Δ*P* of 168 Pa. The production of bimodal filter structures is also possible by blowing the melt polymers at different melting temperatures or blowing the melt polymers with different melt flow index values. As an example, Deng et al.^20^ obtained a multiscale micro-/nanofiber membrane based on polypropylene and polystyrene by a one-step melt-blown technique that exhibited a filtration efficiency of 99.87% and Δ*P* of 37.73 Pa. As a new approach, recently, bioelectrets in bimodal micro-/nanoscale PLA nanofibers were produced by a parallel electrospinning method.^24^ The incorporation of bioelectret bonelike nanocrystalline hydroxyapatite (HABE) into PLA fibers created an orderly alignment under the applied electric field and enhanced the charging capacity and surface potential. Additionally, the filtration efficiency for PM_0.3_ particles at an airflow of 32 L/min was enhanced from 59.38 to 94.38% after adding 30 wt % HABE.

The nanofibers/nanonet structure is an alternative filter structure for highly efficient filtration, particularly for < PM2.5. These structures exhibit a kind of bimodal nature, with fiber diameters clustering around <0.5 and <50 nm.^25^ The low Δ*P*, characteristic of these structures, enhances both energy efficiency and the longer-term use of the filters. The electrospinning method stands out as a fundamental approach to produce nanonets. By precisely controlling the solution and process parameters, nanofiber-nanonet structures of PVDF, PA, PAN, and PU have been produced, and among them, PVDF-based bimodal filters demonstrated a remarkable filtration efficiency of 99.998% due to their electret properties.^8^ The superior performance of PVDF was attributed to its electret properties. Additionally, the addition of surfactants (e.g., sodium dodecyl benzenesulfonate) to the PVDF solution and optimization of the relative humidity of the environment have been reported to facilitate the formation of PVDF nanonets.^26^

In this study, the production of bimodal nanofiber/nanonet filters was achieved using a polymer blend, where one of the components is a sacrificial polymer. PVDF and polyethylene glycol (PEG) (sacrificial) polymers were dissolved at different weight ratios (70:30, 50:50, and 30:70 wt %) and shaped into nanofiber mats via electroblowing (EB) for the first time. Although PVDF and PEG polymers are commonly used together in the production of porous membranes,^27–31^ we aimed to produce high-quality air filters based on nanofibrous mats via EB for the first time. The removal of PEG from the bicomponent nanofiber structure through immersion of the produced fibrous mats in a water bath enhanced the porosity and reduced the diameter of the fibers. Additionally, the fibrous structure changed to nanofibrous/nanonet structure samples produced from the solution with the highest PEG content. Specifically, in nanofiber/nanonet structures, we observed an improvement in Δ*P*, reaching 64% while keeping the filtration efficiency nearly the same. By applying corona discharge treatment, we were able to enhance the filtration efficiency and achieve improvements of up to 65% in the quality factor (QF). These results can be considered a revolution, particularly in the production of highly efficient and low Δ*P* fibrous filters.

## Results and Discussion

### Morphology of the Nanofibrous Filter Media

**Figure 1:**
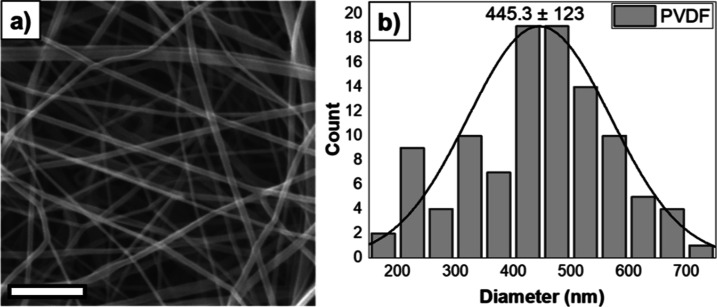
(a) 10 kX magnified SEM image of a neat PVDF nanofibrous mat (scale bar is 5 μm) and (b) its fiber distribution graph.

The scanning electron microscopy (SEM) image and fiber diameter distribution histogram of the PVDF fibrous sample are shown in Figure 1. Accordingly, a structure characterized by dense fibers having an AFD of 445 ± 123 nm without droplets was observed. These observations suggest that the solution concentration and solvent system are well-suited for the EB technique.

**Figure 2:**
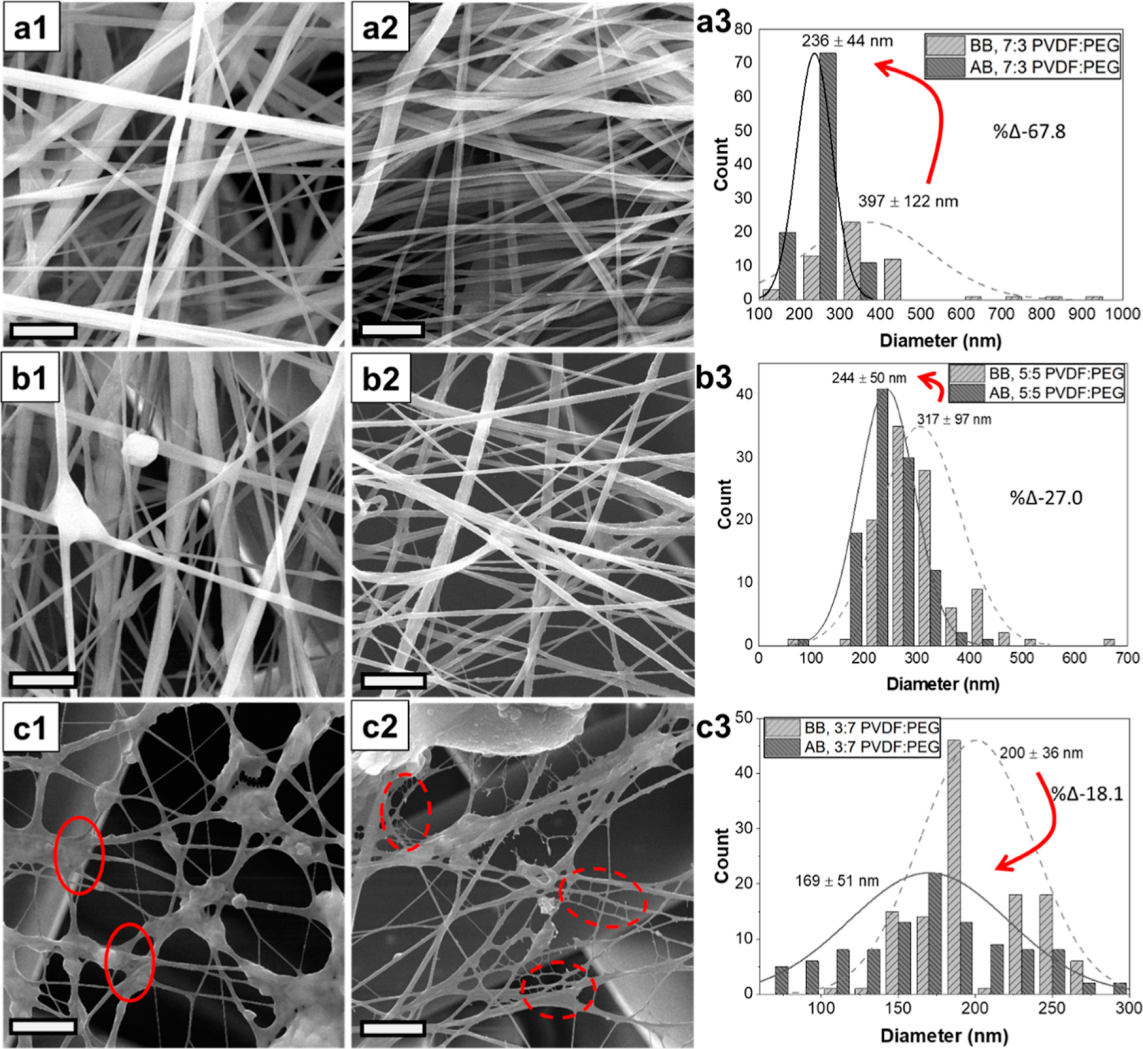
SEM images of (a) PVDF/PEG (7–3), (b) PVDF/PEG (5–5), and (c) PVDF/PEG (3–7) samples (scale bars are 5) and their fiber distribution graphs (BB: before bath, AB: after bath).

According to the SEM images of the PVDF/PEG samples given in Figure 2a–c, for the samples before water bath treatment (Figure 2a1,b1,c1), the fiber diameters decreased from 397 ± 122 to 200 ± 36 nm with increasing PEG ratio in the solutions. Although, with higher PEG content, there was also a noticeable decrease in the fiber diameter distribution (standard deviation values), a significant increase in the density of droplets was observed. These can be attributed to the decrease in solution viscosity due to the increased amount of low-molecular-weight PEG in the solution. In the EB system, the shear forces created by air and electrostatic forces interact with the viscoelastic forces of the polymer solution at the nozzle tip. The interplay between these forces is vital in determining whether a polymer jet forms or droplets form; inadequate viscoelastic forces lead to droplet formation. As given in Table 1, the viscosities of neat PVDF and PVDF/PEG (3–7) solution were measured to be 273.7 and 57.4 mPa·s, respectively. Considering that the viscosity of the polymer solution used in EB should be around 100–5000 mPa·s from our previous studies, increasing the density of the droplet in the PVDF/PEG (3–7) sample was considered to be normal.

**Table 1:** Viscosity of the Prepared Solutions

solution	viscosity (mPa s)
PVDF	273.7
PVDF/PEG (7–3)	169.4
PVDF/PEG (5–5)	114.3
PVDF/PEG (3–7)	57.4

On the other hand, after the water bath treatment (Figure 2a2,b2,c2), the fibers became thinner as PEG was removed from the structure, but the change in the fiber diameter decreased with increasing PEG ratio. The percentage reduction in the fiber diameter for each sample after the water bath treatment was calculated to be 67% for PVDF/PEG (7–3), 27% for PVDF/PEG (5–5), and 18% for the PVDF/PEG (3–7) samples. The PEG removal efficiency through the water bath treatment was also reflected in the percentage mass change of the samples provided in Table 2. Accordingly, the weight loss was 12% for PVDF/PEG (7–3), while it was elevated to 25% for PVDF/PEG (3–7).

**Table 2:** Basis Weight of the Samples

	weight (gsm)			
samples	as produced	after bath	% Δ	after corona
PVDF	2.45			2.45
PVDF/PEG (7–3)	1.86	1.66	–12.0	1.66
PVDF/PEG (5–5)	1.95	1.59	–22.6	1.59
PVDF/PEG (3–7)	1.96	1.56	–25.6	1.56

The most interesting observation in this study is the formation of nanonet-like structures, particularly when the PEG concentration was 70 wt %, as shown in Figure 2c. These nanonet-like structures, with diameters measured to be approximately 45 nm, were concentrated in droplet regions where the fibers coalesce, indicated by the red circles in Figure 2c1. As explained above, increasing PEG content intensified droplet density, resulting in a high PEG content within the droplets. Therefore, the removal of PEG from the structure led to the formation of a nanonet-like structure in these areas.

**Figure 3:**
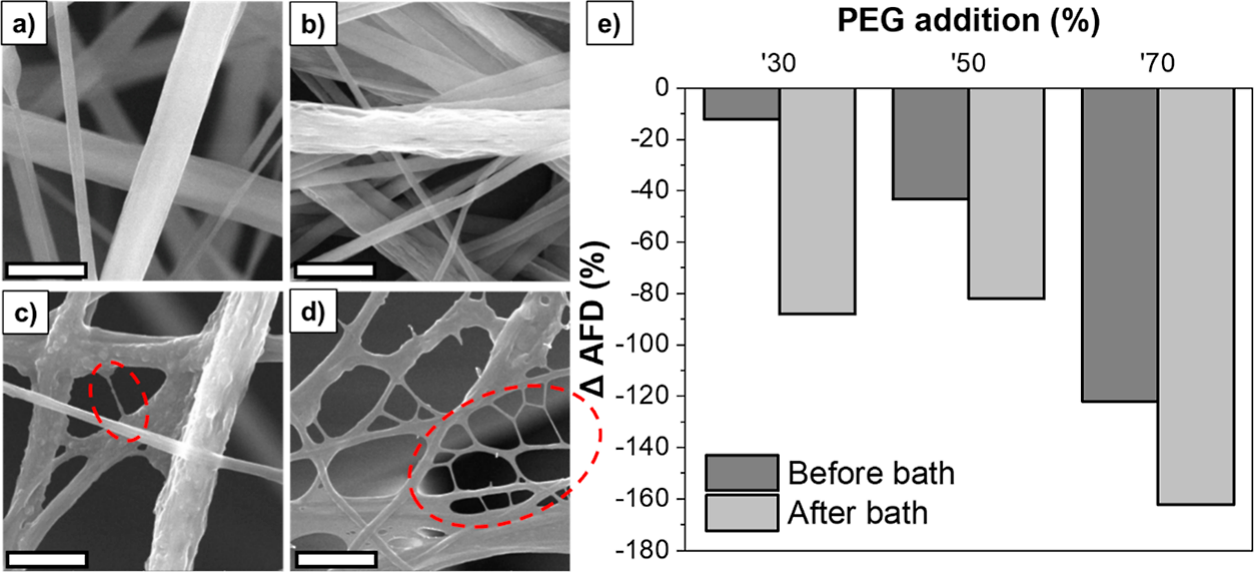
High-magnification SEM images of (a) PVDF and water bath-treated (b) PVDF/PEG (7–3), (c) PVDF/PEG (5–5), and (d) PVDF/PEG (3–7) fibers (scale bars are 2 μm) and (e) % change in fiber diameters compared to neat PVDF as a function of PEG content before and after water bath treatment.

The high-magnification SEM images of the produced fibers were examined to analyze their fiber and surface morphologies, as shown in Figure 3. The pure PVDF sample consists of smooth and cylindrical fibers, whereas the addition of PEG and the subsequent water bath treatment resulted in the loss of cylindrical morphology and smoothness. When the PEG content reached 50 wt %, a nanonet-like structure started to be observed, and the density of these structures reached a maximum at a PEG content of 70 wt %. Since it was not possible to produce fibers from a 100 wt % PEG solution due to its low viscosity, there was no image for the neat PEG sample. Figure 3e presents the percentage change in the AFD of the PVDF–PEG samples before and after water bath treatment compared to the reference PVDF as a function of PEG addition. It is observed that as the PEG content increases, the reduction in fiber diameter increases from 12 to 122% because of lowered solution viscosity. The % reduction in fiber diameter was 162% for the PVDF/PEG (3–7) after water bath treatment.

### Filtration Performance

**Figure 4:**
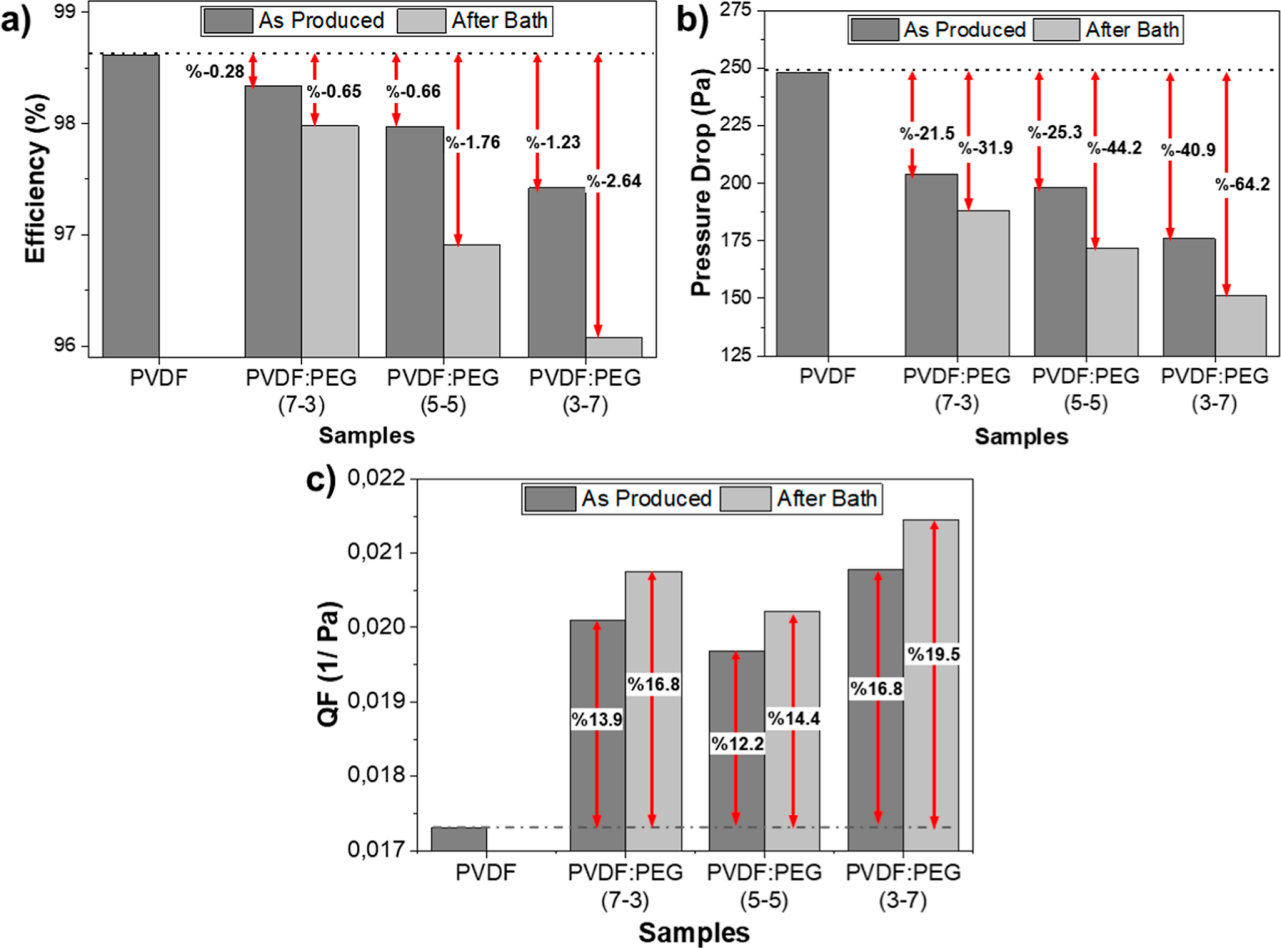
(a) Filtration efficiency, (b) Δ*P*, and (c) QF of the samples before and after water bath treatment.

The filtration efficiency (*n*), Δ*P*, and QF values of the samples are listed in Table 3. Since the neat PVDF mat exhibited a lower porosity associated with thicker and denser fibers, the highest filtration efficiency was obtained from this sample. Filtration efficiency was decreased for all PVDF–PEG samples with increasing PEG content. However, as shown in Figure 4a, the change is very small, where it was a maximum of 1.23% for the PVDF/PEG (3–7). The lowered efficiency can be explained by the finer fibrous structure and increased porosity, which allow increased particle passage through the sample. On the other hand, both PEG addition and water bath treatment also improved the Δ*P* for all samples. Compared with pure PVDF, as shown in Figure 4b, the maximum improvement (64%) was obtained from PVDF/PEG (3–7). The QF, which is inversely related to filtration efficiency and Δ*P*, was calculated for all samples and is shown in Figure 4c. Accordingly, there was an enhancement in the QFs with increasing PEG content. Furthermore, when comparing the samples before and after water bath treatment, an improvement in QF for all samples was also observed because of a greater reduction in Δ*P*. Among the samples, the PVDF/PEG (3–7) sample, which had the lowest Δ*P* value, demonstrated the highest QF with nearly 20%.

**Table 3:** Filtration Performance, Δ*P*, and QF of the Samples As Produced and after Water Bath Treatment

	efficiency (%)		Δ*P* (Pa)		Knudsen number		QF (1/Pa)	
samples	as produced	after bath	as produced	after bath	as produced	after bath	as produced	after bath
PVDF	98.62		248.00		1.46		0.0173	
PVDF/PEG (7–3)	98.34	97.98	204.00	188.00	1.64	2.75	0.0201	0.0208
PVDF/PEG (5–5)	97.97	96.91	198.00	172.00	2.09	2.66	0.0197	0.0202
PVDF/PEG (3–7)	97.42	96.08	176.00	151.00	3.24	3.83	0.0208	0.0215

**Figure 5:**
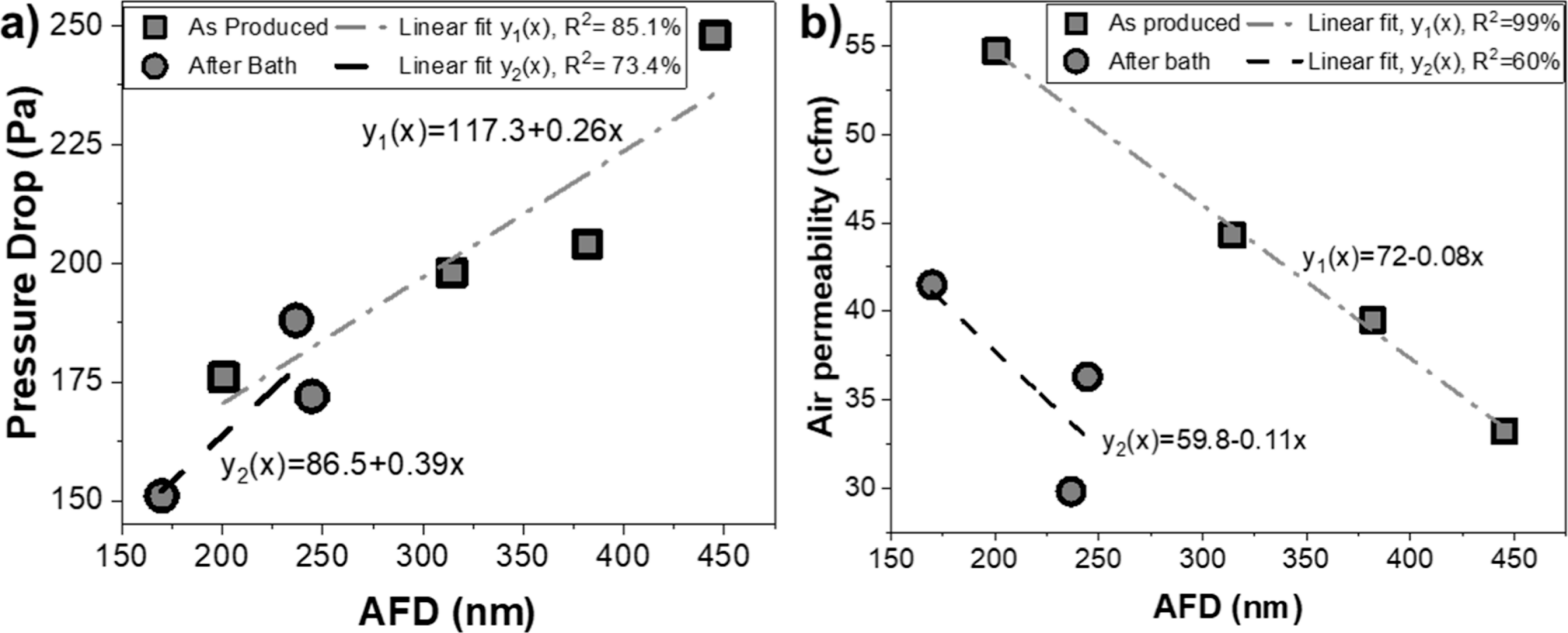
(a) Change of Δ*P* and (b) air permeability as a function of AFD.

In Figure 5a, there is a direct relation found between AFD and Δ*P* for the fibers before and after water bath treatment, which is evident as the reduction in Δ*P* can be attributed to the reduction in the fiber diameter. It is well-established that finer fibers contribute to Δ*P* through the slip effect in air filtration.^32^ This slip effect arises from the nonzero airflow velocity at the surface of individual fibers, known as the slip flow phenomenon. The flow regime of the gas can be determined by relating the mean free path of air molecules to the diameter of nanofibers, which is described by the Knudsen number. There are four flow regimes based on *K*_*n*_ values: continuum flow (*K*_*n*_ ≤ 0.001), slip flow (0.001 < *K*_*n*_ ≤ 0.1), transition flow (0.1 < *K*_*n*_ ≤ 10), and free molecular flow (*K*_*n*_ > 10).^33^ The calculated *K*_*n*_ values for our samples are presented in Table 3. Consequently, our samples exhibit a filtration characteristic known as the transfer regime. Additionally, as shown in Figure 5b, the decrease in the fiber diameters enhances the air permeability of the samples, which can be committed as an increase in the overall % porosity as a result of finer fibers. In Figure 5a,b, there was a small difference in the slopes of the linear fitting curves of Δ*P* and air permeability for the samples before and after water bath treatment. This difference could be a result of the further increased % porosity of the samples after PEG removal. Overall, the decrease in fiber diameters enhances the overall % porosity, leading to a positive contribution to Δ*P* and air permeability along with the slip effect.^34^

**Figure 6:**
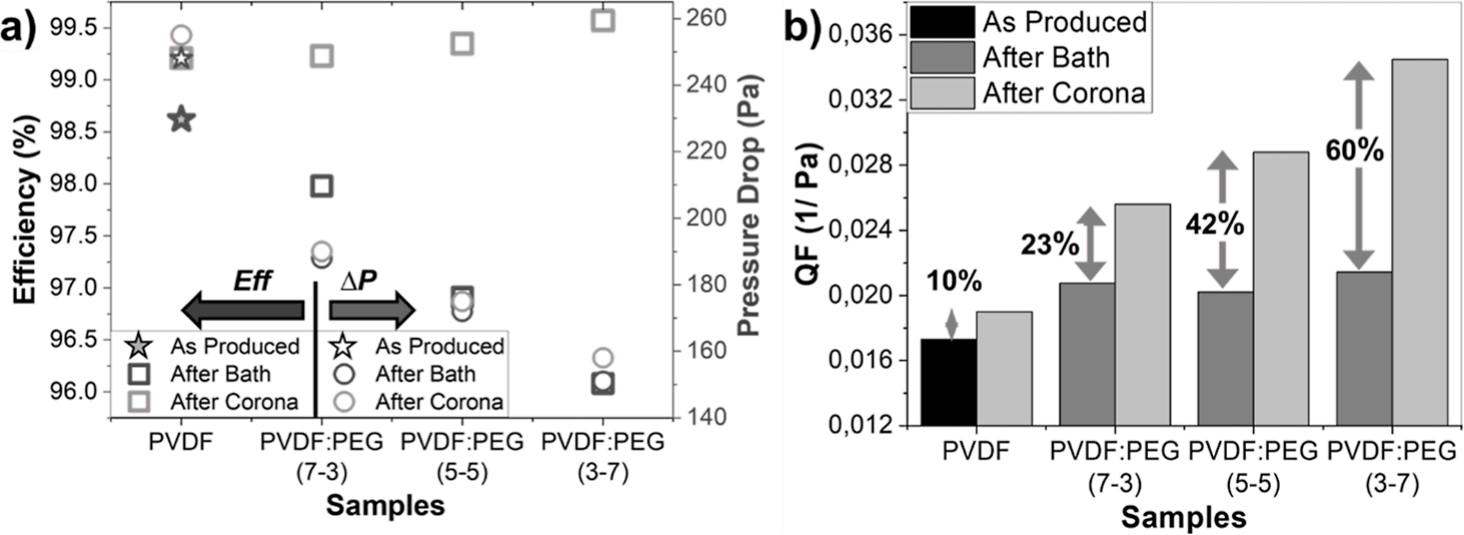
Comparison of the (a) filtration efficiency and Δ*P* and (b) QF of the samples after water bath and corona discharge treatments.

The filtration efficiency of a filter is influenced by various mechanisms such as sieving, straining, interception, diffusion, inertial impaction, and electrostatic capturing. Among these mechanisms, the electrostatic effect plays a significant role and exhibits a superior effect on efficiency.^35^ The effect of the corona discharge process on the filtration performance of the samples is listed in Figure 6a. Accordingly, while the corona discharge process had a negligible effect on Δ*P*, it created a remarkable improvement in the filtration efficiency. Since it is well-known that the corona treatment does not induce any significant changes in fiber morphology, the improvement in filtration efficiency is merely induced by induced electric charges on the fibers. Upon analysis of Figure 6b, it can be observed that the QF increased by 10% in the PVDF, 23% in the PVDF/PEG (7–3), 42% in the PVDF/PEG (5–5), and 60% in the PVDF/PEG (3–7) samples when they were subjected to corona discharging. This improvement in the QF across all samples is directly attributed to the enhancement in filtration efficiency. When particles approach the charged filter surface, they experience stronger electrostatic forces, leading to increased penetration. This is supported by the measurement of the electrostatic surface potential value of these samples following the corona discharge process (Table 4). The PVDF/PEG (3–7), composed of thinner PVDF fibers with a nanonet-like structure, is believed to maximize the retention of charge.

**Table 4:** Measured Electrostatic Surface Potential of the Samples after Corona Discharge Treatment

samples	electrostatic surface potential (V)
PVDF	256.67 ± 53.12
PVDF/PEG (7–3)	436.67 ± 41.10
PVDF/PEG (5–5)	703.33 ± 86.54
PVDF/PEG (3–7)	936.67 ± 134.74

Two of the most crucial parameters influencing the surface potential values of fibrous mats are their pore structures and electrical resistance of the fibrous mats. Accordingly, increasing pore size leads to higher capacity, and increased porosity and resistance result in higher surface potential.^36^ In our study, thinner fibers were obtained with the addition of PEG, and upon the removal of PEG, the fusion point regions disappeared, causing the mat’s electrical resistance to increase. Additionally, with the removal of PEG, further thinning of fibers led to an increased pore size and porosity in the mat. Therefore, as shown in Table 4, higher surface potential values were obtained as the amount of PEG in the samples increased.

**Figure 7:**
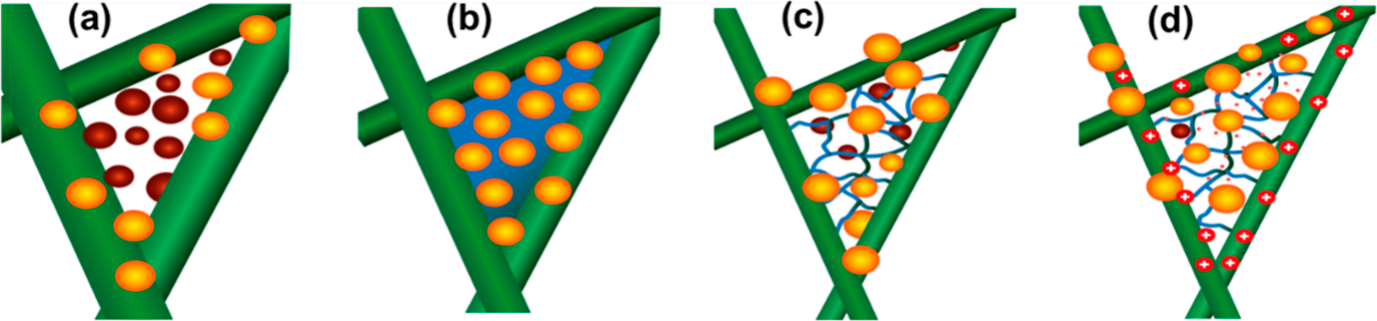
Schematic presentation of the filtration mechanism and nanonet formation models for the samples: (a) only PVDF, (b) PEG–PVDF, (c) after removal of PEG, and (d) after corona discharge treatment samples.

The differences in filtration performances are explained by using the schematic given in Figure 7. Accordingly, in filter samples composed solely of nanofibers, small-sized particles easily pass through the gaps between the fibers compared to PVDF–PEG samples, in which these gaps were filled with either droplets or nanonet structures (Figure 7a). Although it is expected to have a low filtration efficiency for neat PVDF fibrous mats, this effect was not clearly visible since our reference sample had a higher basis weight. Fibrous mats with finer fibers produced from low-viscosity solutions containing a higher PEG ratio have resulted in higher porosity and increased slip effect, causing a lower filtration efficiency and pressure drop. On the other hand, with the addition of PEG higher than 50 wt %, the fusion points of the close fibers were filled with the droplets, as shown in Figure 7b. Although the filled areas are expected to block the particles in airflow and increase the efficiency, the percentage of these areas to the overall structure was very limited; therefore, the expected enhancement could not be observed. As shown in Figure 7c, the removal of the PEG in these regions through the water bath treatment led to a nanonet-like structure behind and also finer nanofibers resulting in higher permeability. While this led to a minor loss in filtration efficiency, a significant improvement in pressure drop was provided. Finally, in samples with an even lower basis weight compared to the reference PVDF, the nanonet structure increased the surface potential of fibers after corona discharge treatments due to the increased total surface area, providing a more effective particle capture and a higher QF of up to 60% (Figure 7d).

## Conclusions

In this study, we successfully produced nanofibrous mats using the EB method from PVDF–PEG blend solutions. Although increased PEG content resulted in droplets in the fibrous mats due to lowered viscosity, finer fibers were obtained with increasing PEG. Additionally, the removal of the PEG polymer through the water bath treatment resulted in thinner and more porous fibers. A nanonet-like structure with a clustered AFD of less than 50 nm in the fusion points of the closest fibers was obtained from the PVDF/PEG (3–7) sample after PEG removal. Although there was a slight decrease in filtration efficiency for the PVDF–PEG samples and after PEG removal, the samples exhibited significantly improved Δ*P* characteristics due to their porous surfaces and bimodal fiber diameters. To enhance the filtration efficiency, corona discharge treatment was applied to all samples, and the PVDF/PEG (3–7) sample demonstrated the highest filtration efficiency of 99.57% and an Δ*P* of 158 Pa, resulting in the highest QF of 0.0345.

## Experimental Section

### Materials and Methods

PVDF (*M*_w_: 477,000 g/mol) and PEG (*M*_w_: 10,000 g/mol) powders were obtained from Arkema Chemicals (Kynar Flex 2801-00) and Merck, respectively. Dimethyl sulfoxide (DMSO, with a purity of 99.8%, Merck) and acetone (with a purity of 99.5%, ISOlab) were used as solvents. Triton X-100 was used as a surfactant and was obtained from Merck. The fibers were collected onto 13 g of polyester (PET) spun-bond fabrics supplied by Mogul Company.

First, 18 wt % PEG and 18 wt % PVDF solutions were separately prepared in an acetone/DMSO mixture with a ratio of 30/70 wt by magnetic stirring at 70 °C for 8 h. Following that, the polymer solutions with PVDF/PEG ratios of 7:3, 5:5, and 3:7 were obtained by mixing the previous solutions in calculated amounts. To provide homogeneity, the solutions were further stirred for 4 h at 70 °C.

**Scheme 1:**
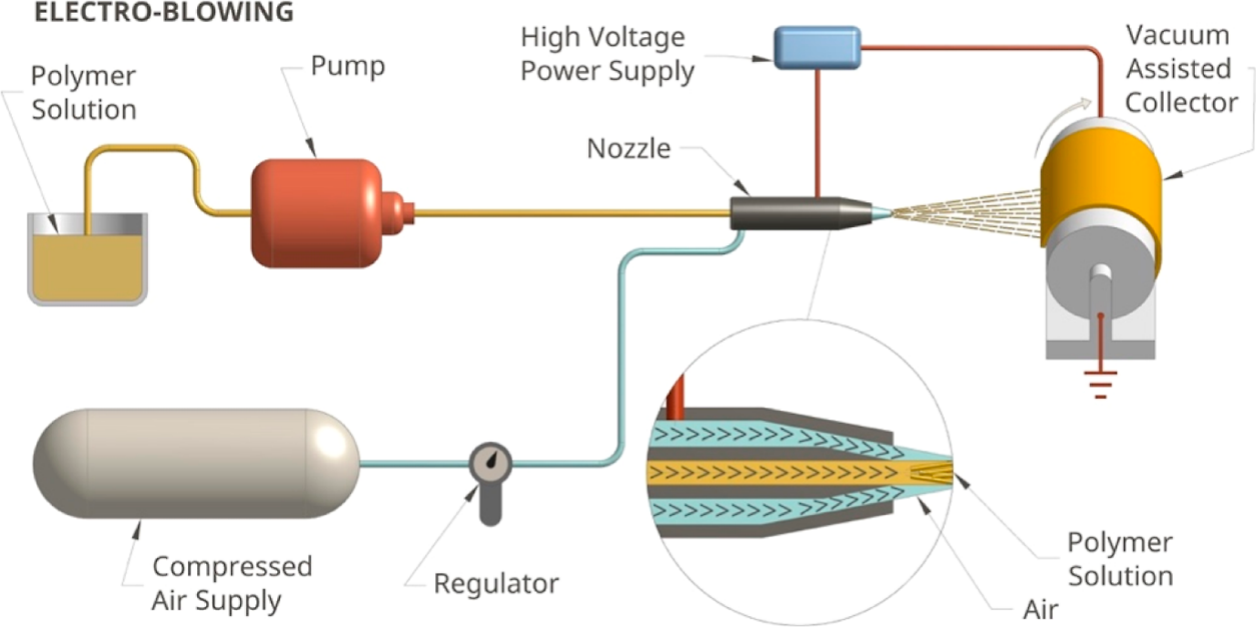
Schematic Presentation of the EB Device

An EB device (AeroSpinner, Areka Ltd.) was utilized to produce all nanofibrous webs. In Scheme 1, the schematic of the device is given. The device consists of a compressed air tank connected to a regulator, a high-voltage power supply, a syringe pump, a coaxial spinning nozzle placed on a homogenizer shaft to provide a homogeneous accumulation of the fibers, and a vacuum-assisted rotating collector with a surface area of 30*20 cm^2^.

All samples were produced at a feeding rate of 10 mL/h, under 1 bar air pressure and 30 kV electric field, with a collector nozzle distance of 30 cm. During production, both the collector and homogenizer rotation speeds were fixed at 40 rpm. The production continued until the collected fibers reached approximately the same basis weight (gsm) value. The final gsm value of the samples is given in Table 2. To compare the effect of blending polymer solutions on the morphology of nanofibrous webs, a reference neat PVDF sample was produced from 18 wt % PVDF solution. Although we tried to produce a reference neat PEG sample, we were not successful due to its low viscosity.

**Scheme 2:**
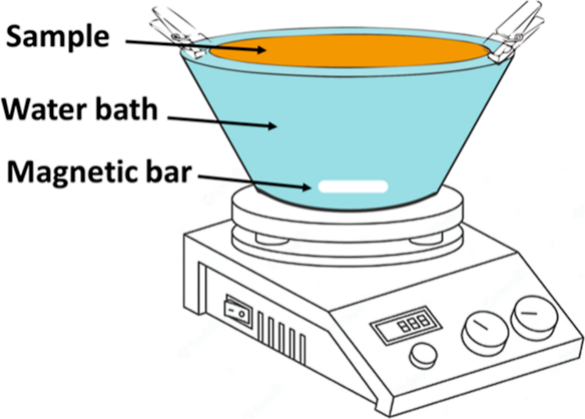
Schematic Presentation of the Bath Treatment of the PVDF/PEG Blend Nanofibrous Mats

A water–2 wt % Triton X-100 bath was prepared to remove PEG components from the blend filter structures, and the samples were placed in the bath, as shown in Scheme 2, on a magnetic stirrer and left for 4 h. The water bath was heated during the treatment on the hot plate set at 100 °C. PEG is a hydrophilic polymer that can dissolve quickly in water. It was presumed that PEG components present on the surface of the fibers would dissolve easily in the presence of only water. However, to facilitate the dissolution of the PEG components mixed with hydrophobic PVDF, a surfactant was added to the bath.

Corona charging was performed using a negative corona discharge device (Chargemaster 5, Simco-Ion). For the corona application, the device’s electrode was positioned approximately 4 cm above a rotating drum. Nanofiber samples were placed on the drum, which was rotating at a speed of 23 rpm, and charged for 5 min under a charging voltage of 20 kV.

### Characterization

The viscosities of all prepared solutions were measured with a rotational viscometer (Fungilab, α Series) before fiber production and are presented in Table 1. The morphologies of the fibrous structures were examined using a scanning electron microscope (TESCAN VEGA 3). Prior to SEM analysis, a 10 nm-thick gold/palladium coating was sputtered onto the samples to ensure conductivity. AFDs and standard deviations were calculated using measurements taken from SEM images at magnifications of 5 and 20 kX and from the fibers excluding the nanonet structures.

An automatic filter testing device (8130A model, TSI Inc.) was used to evaluate the filtration performance of the samples in terms of Δ*P* and filtration efficiency [η]. Solid salt particles with a diameter of 0.26 ± 0.07 μm were generated from a NaCl solution (2% by weight). Nanofibrous mats with an effective area of 100 cm^2^ were tested against NaCl aerosols at a face velocity of 15.83 cm/s. Filtration efficiency (η) was calculated using eq 1
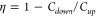
1where *C*_down_ represents the downstream particle concentration and *C*_up_ is the upstream particle concentration. The mathematical expression of the QF, which evaluates the quality of the filter sample by considering both filtration efficiency and Δ*P*, is given in eq 2
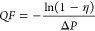
2

The Knudsen number (*K*_*n*_) is used to describe the molecular movements of air molecules near the surface of fibers and can be calculated by using eq 3. Here, λ represents the mean free path of the gas molecules, which is equal to 65 nm at a temperature of 298 K and a pressure of 1 atm. *d*_f_ represents the diameter of the fibers.^32^
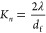
3

The FMX-004 electrostatic field meter device (Simco-Ion) was utilized to measure the surface potential of the samples after corona charging. Measurements were taken from different regions of the samples to determine the average value of the surface potential.

## References

[ref1] He M.; Ichinose T.; Kobayashi M.; Arashidani K.; Yoshida S.; Nishikawa M.; Takano H.; Sun G.; Shibamoto T. Differences in Allergic Inflammatory Responses between Urban PM2.5 and Fine Particle Derived from Desert-Dust in Murine Lungs. Toxicol. Appl. Pharmacol. 2016, 297, 41–55. 10.1016/j.taap.2016.02.017. 26917405

[ref2] Liu Y.; Wang X.; Li N.; Wang X.; Shi L.; Wu E.; Wang R.; Shan M.; Zhuang X. UV-Crosslinked Solution Blown PVDF Nanofiber Mats for Protective Applications. Fibers Polym. 2020, 21 (3), 489–497. 10.1007/s12221-020-9666-5.

[ref3] Satsangi G. S.; Lakhani A.; Khare P.; Singh S. P.; Kumari K. M.; Srivastava S. S. Measurements of Major Ion Concentration in Settled Coarse Particles and Aerosols at a Semiarid Rural Site in India. Environ. Int. 2002, 28 (1–2), 1–7. 10.1016/S0160-4120(01)00122-2. 12046945

[ref4] Kunjuzwa N.; Nthunya L. N.; Nxumalo E. N.; Mhlanga S. D. Chapter 5 - The Use of Nanomaterials in the Synthesis of Nanofiber Membranes and Their Application in Water Treatment. In Advanced Nanomaterials for Membrane Synthesis and its Applications; Lau W.-J., Ismail A. F., Isloor A., Al-Ahmed A., Eds.; Micro and Nano Technologies; Elsevier, 2019, pp 101–125.

[ref5] Galka N.; Saxena A. High Efficiency Air Filtration: The Growing Impact of Membranes. Filtr. Sep. 2009, 46 (4), 22–25. 10.1016/S0015-1882(09)70157-0.

[ref6] Grafe T.; Graham K. Polymeric Nanofibers and Nanofiber Webs: A New Class of Nonwovens. Int. Nonwovens J. 2003, os-12 (1), 1558925003os 10.1177/1558925003os-1200113.

[ref7] Jung S.; Kim J. Advanced Design of Fiber-Based Particulate Filters: Materials, Morphology, and Construction of Fibrous Assembly. Polymers 2020, 12 (8), 1714 10.3390/polym12081714. 32751674 PMC7464808

[ref8] Liu H.; Zhang S.; Liu L.; Yu J.; Ding B. High-Performance PM0.3 Air Filters Using Self-Polarized Electret Nanofiber/Nets. Adv. Funct. Mater. 2020, 30 (13), 1909554 10.1002/adfm.201909554.

[ref9] Leung W. W.-F.; Choy H.-F. Transition from Depth to Surface Filtration for a Low-Skin Effect Filter Subject to Continuous Loading of Nano-Aerosols. Sep. Purif. Technol. 2018, 190, 202–210. 10.1016/j.seppur.2017.08.060.

[ref10] Zhang J.; Chen G.; Bhat G. S.; Azari H.; Pen H. Electret Characteristics of Melt-Blown Polylactic Acid Fabrics for Air Filtration Application. J. Appl. Polym. Sci. 2020, 137 (4), 48309 10.1002/app.48309.

[ref11] Borojeni I. A.; Gajewski G.; Riahi R. A. Application of electrospun nonwoven fibers in air filters. Fibers 2022, 10, 15 10.3390/fib10020015.

[ref12] Eun J.; Lee H.; Jeon S. Regeneration of an Electret Filter by Contact Electrification. RSC Adv. 2021, 11 (8), 4610–4615. 10.1039/D0RA09769A. 35424378 PMC8694494

[ref13] Wang C.; Song X.; Li T.; Zhu X.; Yang S.; Zhu J.; He X.; Gao J.; Xu H. Biodegradable Electroactive Nanofibrous Air Filters for Long-Term Respiratory Healthcare and Self-Powered Monitoring. ACS Appl. Mater. Interfaces 2023, 15 (31), 37580–37592. 10.1021/acsami.3c08490. 37490285

[ref14] Wang Z.; Chen G.; Hong X.; Yu J.; Zhang J.; Ding Y.; Lou Q.; He H. Study on Charge Characteristic of Melt-Blown Polypropylene Electret Fabric by Hydrocharging Technique. J. Electrost. 2022, 116, 103683 10.1016/j.elstat.2022.103683.

[ref15] Wang N.; Cai M.; Yang X.; Yang Y. Electret Nanofibrous Membrane with Enhanced Filtration Performance and Wearing Comfortability for Face Mask. J. Colloid Interface Sci. 2018, 530, 695–703. 10.1016/j.jcis.2018.07.021. 30015155

[ref16] Xiao Y.; Wang Y.; Zhu W.; Yao J.; Sun C.; Militky J.; Venkataraman M.; Zhu G. Development of Tree-like Nanofibrous Air Filter with Durable Antibacterial Property. Sep. Purif. Technol. 2021, 259, 118135 10.1016/j.seppur.2020.118135.

[ref17] Bui T. T.; Shin M. K.; Jee S. Y.; Long D. X.; Hong J.; Kim M.-G. Ferroelectric PVDF Nanofiber Membrane for High-Efficiency PM0.3 Air Filtration with Low Air Flow Resistance. Colloids Surf. A Physicochem. Eng. Asp. 2022, 640, 128418 10.1016/j.colsurfa.2022.128418. 35125661 PMC8800002

[ref18] Karabulut F. N. H.; Höfler G.; Chand N. A.; Beckermann G. W. Electrospun Nanofibre Filtration Media to Protect against Biological or Nonbiological Airborne Particles. Polymers 2021, 13 (19), 3257 10.3390/polym13193257. 34641073 PMC8511993

[ref19] Liu J.; Ding C.; Dunne F. O.; Guo Y.; Fu X.; Zhong W.-H. A Bimodal Protein Fabric Enabled via In Situ Diffusion for High-Performance Air Filtration. Environ. Sci. Technol. 2020, 54 (19), 12042–12050. 10.1021/acs.est.0c02828. 32936622

[ref20] Deng N.; He H.; Yan J.; Zhao Y.; Ben Ticha E.; Liu Y.; Kang W.; Cheng B. One-Step Melt-Blowing of Multi-Scale Micro/Nano Fabric Membrane for Advanced Air-Filtration. Polymer 2019, 165, 174–179. 10.1016/j.polymer.2019.01.035.

[ref21] Przekop R.; Gradoń L. Deposition and Filtration of Nanoparticles in the Composites of Nano- and Microsized Fibers. Aerosol Sci. Technol. 2008, 42 (6), 483–493. 10.1080/02786820802187077.

[ref22] Zhang S.; Tang N.; Cao L.; Yin X.; Yu J.; Ding B. Highly Integrated Polysulfone/Polyacrylonitrile/Polyamide-6 Air Filter for Multilevel Physical Sieving Airborne Particles. ACS Appl. Mater. Interfaces 2016, 8 (42), 29062–29072. 10.1021/acsami.6b10094. 27700022

[ref23] Gungor M.; Toptas A.; Calisir M. D.; Kilic A. Aerosol Filtration Performance of Nanofibrous Mats Produced via Electrically Assisted Industrial-Scale Solution Blowing. Polym. Eng. Sci. 2021, 61 (10), 2557–2566. 10.1002/pen.25780.

[ref24] Tang M.; Jiang L.; Wang C.; Li X.; He X.; Li Y.; Liu C.; Wang Y.; Gao J.; Xu H. Bioelectrets in Electrospun Bimodal Poly(Lactic Acid) Fibers: Realization of Multiple Mechanisms for Efficient and Long-Term Filtration of Fine PMs. ACS Appl. Mater. Interfaces 2023, 15 (21), 25919–25931. 10.1021/acsami.3c02365. 37192220

[ref25] Robert B.; Nallathambi G. A Concise Review on Electrospun Nanofibres/Nanonets for Filtration of Gaseous and Solid Constituents (PM2.5) from Polluted Air. Colloid Interface Sci. Commun. 2020, 37, 100275 10.1016/j.colcom.2020.100275.

[ref26] Li X.; Wang C.; Huang X.; Zhang T.; Wang X.; Min M.; Wang L.; Huang H.; Hsiao B. S. Anionic Surfactant-Triggered Steiner Geometrical Poly(Vinylidene Fluoride) Nanofiber/Nanonet Air Filter for Efficient Particulate Matter Removal. ACS Appl. Mater. Interfaces 2018, 10 (49), 42891–42904. 10.1021/acsami.8b16564. 30427661

[ref27] Fadaei A.; Salimi A.; Mirzataheri M. Structural Elucidation of Morphology and Performance of the PVDF/PEG Membrane. J. Polym. Res. 2014, 21 (9), 545 10.1007/s10965-014-0545-x.

[ref28] Ma W.; Rajabzadeh S.; Shaikh A. R.; Kakihana Y.; Sun Y.; Matsuyama H. Effect of Type of Poly(Ethylene Glycol) (PEG) Based Amphiphilic Copolymer on Antifouling Properties of Copolymer/Poly(Vinylidene Fluoride) (PVDF) Blend Membranes. J. Membr. Sci. 2016, 514, 429–439. 10.1016/j.memsci.2016.05.021.

[ref29] Parshetti G. K.; Doong R. Dechlorination of Trichloroethylene by Ni/Fe Nanoparticles Immobilized in PEG/PVDF and PEG/Nylon 66 Membranes. Water Res. 2009, 43 (12), 3086–3094. 10.1016/j.watres.2009.04.037. 19476967

[ref30] Wang L.-Y.; Yong W. F.; Yu L. E.; Chung T.-S. Design of High Efficiency PVDF-PEG Hollow Fibers for Air Filtration of Ultrafine Particles. J. Membr. Sci. 2017, 535, 342–349. 10.1016/j.memsci.2017.04.053.

[ref31] Zuo D.; Xu Y.; Xu W.; Zou H. The Influence of Peg Molecular Weight on Morphologies and Properties of Pvdf Asymmetric Membranes. Chin. J. Polym. Sci. 2008, 26 (04), 405–414. 10.1142/S0256767908003072.

[ref32] Choi H.-J.; Kumita M.; Seto T.; Inui Y.; Bao L.; Fujimoto T.; Otani Y. Effect of Slip Flow on Pressure Drop of Nanofiber Filters. J. Aerosol Sci. 2017, 114, 244–249. 10.1016/j.jaerosci.2017.09.020.

[ref33] Zhao X.; Wang S.; Yin X.; Yu J.; Ding B. Slip-Effect Functional Air Filter for Efficient Purification of PM 2.5. Sci. Rep. 2016, 6 (1), 35472 10.1038/srep35472. 27748419 PMC5066256

[ref34] Yilmaz N. D.; Banks-Lee P.; Powell N. B.; Michielsen S. Effects of Porosity, Fiber Size, and Layering Sequence on Sound Absorption Performance of Needle-Punched Nonwovens. J. Appl. Polym. Sci. 2011, 121 (5), 3056–3069. 10.1002/app.33312.

[ref35] Kilic A.; Russell S.; Shim E.; Pourdeyhimi B. 4—The Charging and Stability of Electret Filters. In Fibrous Filter Media; Brown P. J., Cox C. L., Eds.; Woodhead Publishing Series in Textiles; Woodhead Publishing, 2017, pp 95–121.

[ref36] Liang M.; Hébraud A.; Schlatter G. Modeling and On-Line Measurement of the Surface Potential of Electrospun Membranes for the Control of the Fiber Diameter and the Pore Size. Polymer 2020, 200, 122576 10.1016/j.polymer.2020.122576.

